# Prevalence of national treatment algorithm defined smear positive pulmonary tuberculosis in HIV positive patients in Brazzaville, Republic of Congo

**DOI:** 10.1186/1756-0500-7-578

**Published:** 2014-08-28

**Authors:** Laure Stella Ghoma Linguissi, Pembe Issamou Mayengue, Anissa Sidibé, Jeannhey C Vouvoungui, Mitawa Missontsa, Igor Kevin Madzou-Laboum, Gaston Bango Essassa, Sunny Oyakhirome, Matthias Frank, Veronique Penlap, Francine Ntoumi

**Affiliations:** Fondation Congolaise pour la Recherche Médicale, Brazzaville, Republic of Congo; Faculté des Sciences de la Santé, Université Marien Ngouabi, Brazzaville, PB 2672 Republic of Congo; Institute for Tropical Medicine, University of Tübingen, Tübingen, Germany; Centre Antituberculeux de Brazzaville, Programme de Lutte contre la Tuberculose, Brazzaville, Republic of Congo; University Yaoundé1, Yaoundé, Cameroon; German Center for Infection Research (DZIF), Braunschweig, Germany

**Keywords:** Tuberculosis, Microscopy, Pulmonary TB, HIV infection, Republic of Congo

## Abstract

**Background:**

In the Republic in Congo, the national algorithm for the diagnosis of pulmonary tuberculosis (TB) relies on Ziehl-Neelsen (ZN) sputum smear microscopy, chest X-ray radiography (CXR) and clinical symptoms. Microscopy positive pulmonary TB (MPT+) is defined as symptoms of TB and a positive ZN smear. Microscopy negative pulmonary TB (MPT-) is defined as symptoms of TB, a negative ZN smear but CXR changes consistent with TB. The present cross-sectional study was designed to determine the prevalence of positive and negative MPT individuals among HIV positive and HIV negative individuals presenting to an ambulatory TB treatment center (CTA) in Brazzaville.

**Methods:**

All study participants underwent a physical examination, chest radiography and three ZN sputum smear examinations and HIV testing. Viral load and CD4 counts were determined for HIV positive individuals.

**Results:**

775 individuals presented with symptoms of TB. 425 individuals accepted the voluntary HIV test. 133 (31.3%) were HIV positive (HIV+) and 292 (68.7%) were HIV negative (HIV-). Of the 292 HIV- individuals 167 (57%) were classified as positive MPT and 125 (43%) as negative MPT. Of the 133 HIV positive individuals 39 (29%) were classified as MPT + and 94 (71%) as MPT-.

**Conclusion:**

Our study shows that the prevalence of positive MPT individuals is lower among HIV positive individuals compared to HIV negative individuals in agreement to reports from other countries. The data suggest that a substantial number of HIV positive pulmonary TB cases are not detected by the national algorithm and highlight the need for new diagnostic tests in this population.

## Background

There are approximately 34 million people currently living with HIV, one-third of these is estimated to also be infected with tuberculosis (TB) [1–3]. Although antiretrovirals (ARV) can reduce the risk of death in HIV infected individuals, an untreated TB patient starting this therapy might be exposed to immune-reconstitution inflammatory syndrome (IRIS) which is associated with increased morbidity and potential mortality [4].

In sub-Saharan Africa, TB is often the first manifestation of HIV infection, and it is the leading cause of death among HIV infected patients [5–7]. The countries of the world with the highest HIV prevalence rates are also those with high rates of TB [8], these countries include: Swaziland, Botswana, Lesotho, and South Africa [1, 9]. The highest number of TB/HIV co-infection has been reported in southern Africa, where more than 60% of TB patients are HIV positive [5, 6, 10]. The burden of TB and TB-HIV co-infection is no less alarming in the Republic of Congo. According to the World Health Organization (WHO), in 2011, the incidence of TB in Republic of Congo was 473 per 100,000 population (including TB/HIV co-infections) and 31% of TB patients were HIV positive [11].

The diagnosis of HIV-associated TB is often challenging and nucleic acid amplification tests, including the GeneXpert MTB/RIF assay and urinary LAM assays are known to improve the diagnosis of both smear-negative and extrapulmonary TB in HIV positive individuals [12].

**Figure 1:**
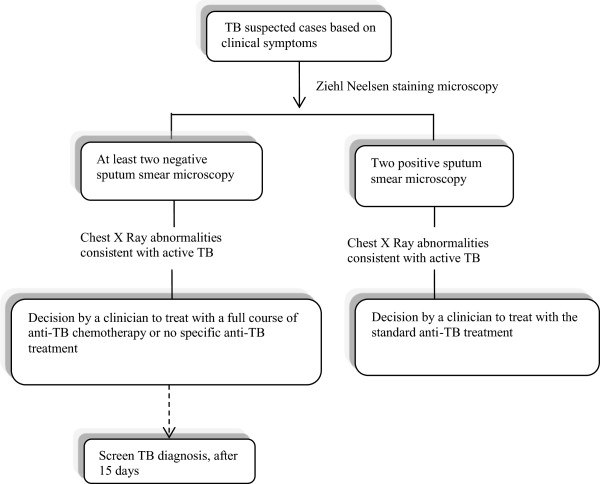
**National algorithm for the diagnosis of pulmonary TB.**

TB diagnosis worldwide currently heavily relies on sputum smear microscopy to detect acid-fast bacilli (AFB), as it is rapid, inexpensive, and highly specific for identifying active pulmonary tuberculosis (PTB) [13, 14]. This technique is known to identify the most cases with excellent specificity, but has an approximate sensitivity of 35% to 70% [15–18]. Indeed, a particular challenge for clinicians is the rising incidence of HIV related TB, often associated with an increase in smear negative microscopy cases [19–22] Unfortunately, the most sensitive techniques available such as mycobacterial culture and nucleic acid amplification techniques, are expensive, resource intensive and thus not routinely available in many African countries [23–27]. Current culture-based methods takes more than two weeks [28] and there is a need for faster diagnosis of TB in HIV- and HIV + patients as well as delays in diagnosis and treatment are associated with high mortality risk [29–32].In the Republic of Congo, the current national guidelines for TB diagnosis relies on sputum smear microscopy, X-ray radiography and clinical symptoms to confirm TB cases (Figure 1). The present study is designed as a diagnostic cross sectional study of HIV + and HIV - patients suspected of having pulmonary TB. We aimed to demonstrate the utility of the national diagnosis algorithm in confirming pulmonary TB in HIV + and HIV -individuals. In addition we assessed virologic and immunologic parameters in the HIV positive individuals. More specifically, we seek to evaluate the need to implement new diagnostic tools to increase the detection rate of TB in HIV+/sputum smear-negative cases.

## Methods

### Selection of patients

The study participants were recruited at the anti-tuberculosis center (CAT) of Brazzaville during the study period from February to June 2011. All consecutive TB suspects regardless of HIV status were eligible for the study. Informed consent was obtained from all study participants or their guardians. Ethical authorization was obtained from the Comité d’éthique Institutionnel de la Recherche en Santé (CERSSA) (No. 00000067/DGRST/CERSSA).

### Specimen collection

Demographic data (age, place of residence) and clinical data were recorded for all participants during a medical interview and a physical examination by a clinician. Chest radiography and sputum collection was performed according to the national diagnostic algorithm. Participants provided three sputum specimens over the course of 2 days. The first specimen was collected when the patient attended the CAT for the first time. A container was given to the participant for collecting an early morning sample at home the next day, and a third was collected again at the center when the patient brought back the early morning specimen. The samples were tested with the direct Ziehl-Neelsen light microscopy method following standard instructions on proper sputum submission. Sputum microscopy was performed at the onsite laboratory, with density of acid fast bacilli graded as 1, 2 or 3 + .

### HIV testing

Blood samples were tested using two rapid tests, Determine HIV 1/2 test (Alere GmbH, Koln, *Germany*) and ImmunoComb II *HIV 1* & 2 BiSpot (*Orgenics*, Yavne, Israel). In case of discordance between these two tests, the enzyme-linked immunosorbent assay (ELISA, Vironostika®HIV-1 Plus O Microelisa System, UK) was performed by the reference lab which is the Laboratoire National de Santé Publique.

### CD4+/CD8+ cells counts

Approximately 5 ml of blood was collected in an EDTA tube for counting T cells. The blood samples were processed immediately within 2 hours of collection, for determination of the absolute counts of CD4+, CD8+ cells by activated cell sorting (FACS) count (*Partec* GmbH, Munster, Germany), following manufacturer’s instructions.

### Quantification of plasma viral load

Quantification of plasma viral load of HIV-1 was done using Biocentric™ kit, on a platform of Roche LightCycler ® 480 using the technique of real-time PCR. A volume of 10 ml obtained after RNA extraction from plasma was used for analysis. The detection limit was 1,500 viral RNA copies / ml (2.3 log copies / ml). The determination of viral load was performed at the Laboratory of Molecular Biology at the Faculty of Health Sciences /University Marien Ngouabi, Brazzaville.

### National diagnosis algorithm (Figure 1)

At the first consultation, all patients suspected of TB were clinically assessed by a trained health professional. The assessment includes screening for clinical signs and symptoms such as current cough (if self-reported cough was > 2 weeks), fever, night sweats and weight loss. Sputum samples were collected and examined using Ziehl-Neelsen staining microscopy. A chest X-ray was performed and HIV testing was offered to all individuals.

### Case definitions

Pulmonary TB (PTB) positive microscopy: Patients with at least 2 positive sputum samples were classified as positive for pulmonary TB. These PTB positive patients were given the standard anti-TB treatment according to National guidelines.

Pulmonary TB negative microscopy: Patients with at least 2 sputum samples negative for AFB and with symptoms of TB, a negative ZN smear but CXR changes consistent with TB were classified as negative for pulmonary TB. These PTB negative patients were treated with a full course of anti-TB chemotherapy if 15 days later the screening is positive.

Patients without pulmonary TB: Individuals with negative sputum samples for AFB and no X-ray changes suggestive of TB.

### Statistical analysis

The demographic, clinical and laboratory results were entered into a database and analyzed using SPSS version 16 (SPSS Inc, Chicago, IL, USA). The mean and standard deviation of the age, CD4 and CD8 T cells and viral load were calculated using student’s t-test for comparison. Logistic regression was used to evaluate the effect of age, gender and clinical parameters on HIV infection. Kruskal-Wallis test was used to compare the proportion of HIV infected patients with different levels of CD4+, CD8+ T cells and viral load between AFB positive and negative microscopy. Differences were considered statistically significant when the p value was ≤ 0.05.

## Results

### Characteristic of patients who consented to the HIV test

**Figure 2:**
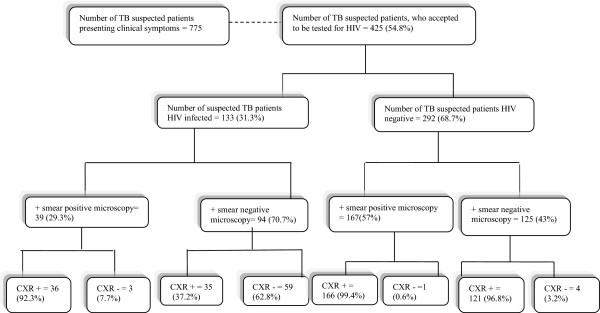
**Sputum smear microscopy, HIV test and chest X-Ray results for the study participants.** + means Patients with smear positive or smear negative microscopy CXR means Chest X-ray.

From February to June 2011, 775 patients attending the consultation service at the TB center of Brazzaville (CAT) were deemed suspected TB cases based on clinical examination (Figure 2). Of these, 425 (54.8%) underwent HIV testing at the time of TB diagnosis specimen collection. Among these, 133 (31.3%) were HIV + and 292 were HIV-.

As presented on Table 1, the mean age of all participants was 34.9 years ±13.01 and most of the patients were older than 25 years. Gender, signs of TB, axillary temperature did not significantly differ according to HIV status.

**Table 1:** **Characteristics of patients (who accepted to do the HIV test) enrolled at the TB Center in Brazzaville**

	All	HIV positive	HIV negative	Crude OR(95%CI)	P-value
Number of patients	425(54.8)	133(31.3)	292	--	--
Age (years)Mean ± SD	34.9 ± 1	38.37 ± 9	33.40 ± 1	--	0.000
Age group					
14-24ans	98(23.7)	9(9.2)	89(90.8)	Baseline	
25-34ans	123(29.7)	33(26.8)	90(73.2)	3.6(1.64-8.01)	0.001
≥ 35ans	193(46.6)	84(43.5)	109(56.5)	7.6(3.63-16.00)	<0.001
Missing	11				
Gender					
Female	177(42.3)	71(40.1)	106(59.9)		
Male	241(57.7)	55(22.8)	186(77.2)	0.4 (0.29-0.67)	<0.001
Missing	7				
Signs of TB					
No	99(23.9)	34(34.3)	65(65.7)		
Yes	319(76.3)	92(28.8)	227(71.2)	0.7(0.48-1.25)	0.298
Missing	7				
Cough					
No	0(0)	0	0		
Yes	418(100)	126(30.1)	292(69.9)	--	--
Missing	7				
Pale skin					
No	355(85.5)	101(28.5)	254(71.5)		
Yes	60(14.5)	25(41.7)	35(58.3)	1.8(1.02-3.15)	0.041
Missing	10				
Axillary temperature >37.5°C					
No	37(8.9)	11(29.7)	26(70.3)		
Yes	379(91.1)	114(30.1)	265(69.9)	1.02(0.48-2.13)	0.965
Missing	9				
Asthenia					
No	38(9.3)	8(20.5)	31(79.5)		
Yes	378(90.6)	117(30.8)	261(69.2)	1.7(0.78-3.89)	0.180
Missing	8				
Anorexia					
No	80(19.1)	17(21.2)	63(78.8)		
Yes	338(80.9)	109(32.2)	229(67.8)	1.7(0.98-3.16)	0.056
Missing	7				
Weight loss					
No	13(3.12)	2(15.4)	11(84.6)		
Yes	404(96.9)	124(30.7)	280(69.3)	2.4(0.53-11.15)	0.251
Missing	8				
Received TB treatment, after diagnosis					
No	61(14.6)	54(88.5)	7(11.5)		
Yes	357(85.4)	72(20.2)	285(79.8)	0.03(0.01-0.07)	<0.001
Missing	7				

Of the 292 HIV- individuals, 167 (57%) were classified as PTM positive and 125 (43%) as PTM negative. Of the 133 HIV + individuals 39 (29.3%) were classified as PTM positive and 94 (70.7%) as PTM negative (Table 2 and Figure 2). Thirty-six (92.3%) and thirty-five (37.2%) AFB positive and negative patients respectively, were positive for X-ray (Figure 2) (<0.001).

**Table 2:** **CD4+ and CD8+ T cells counts in HIV Congolese patients with positive or negative sputum detected by microscopy using Ziehl-Neelsen staining**

	All	Positive microscopy	Negative microscopy	P-value*
Number of HIV-1 patients	133(31.3%)	39(29,3)	94(70.7)	<0.001--
Immunological category (CDC)				
CD4+ T-cell count (cells/mm3, mean ± SD)	289.4 ± 282.9	385.4 ± 278.1	239.6 ± 274.9	0.029
Absolute CD4+ T-cell counts	53(67.1)	12(44.4)	41(78.8)	
<350/mm3	11(13.9)	7(26)	4(7.7)	0.007
350–500/mm3	15(19.0)	8(29.6)	8(13.5)	
>500/mm3	54			
Missing				
CD8+ T-cell counts (cells/mm3, mean ± SD)	850.9 ± 547.4	647.3 ± 555.2	956.6 ± 517.5	0.016
Absolute CD8+ T-cell counts	18(22.7)	4(14.8)	14(26.9)	
<400/mm3	16(20.3)	3(11.1)	13(25)	0.001
400–799/mm3	30(38)	8(29.6)	22(42.3)	
800–1200/mm3	15(19)	12(44.5)	3(5.8)	
>1200/mm3	54			
Missing				
CD4/CD8 ratio (mean ± SD)	0.34 ± 0.51	0.59 ± 0.5	0.25 ± 0.5	0.003
Missing	54			

*: Chi-square Test.

### CD4+/CD8+ T cells, viral load and sputum microscopy examination in HIV patients

Concerning the 133 HIV patients, CD4+ T cells counts have been reported according to the results of sputum microscopy examination in Table 2. Overall mean baseline CD4+ and CD8+ T cell count was 289.4 and 850.9 cells/ml respectively (Table 2). HIV + patients with positive AFB had higher CD4+ counts (385.4 ± 278.1 cells/ml) when compared to those with a negative AFB (239.6 ± 274.9 cells/ml) (p < 0.029). Patients with CD4 cell counts below 350 cells/mm3 were significantly higher among negative AFB patients (p < 0.007). The mean CD4/CD8 ratio was significantly higher in positive smear pulmonary tuberculosis than negative smear (0.59 versus 0.25, P < 0.003).

The mean viral load was similar in AFB positive and AFB negative groups and no significant difference between those with positive and negative AFB (Table 3) was found.

**Table 3:** **Viral load in HIV Congolese patients with sputum smear positive or negative by microscopy stained using Ziehl-Neelsen staining**

	All	Smear positive microscopy	Smear negative microscopy	P-value
Number HIV-1 infected patients	133	39(29,3)	94(70.7)	--
Mean ± SD, log10 copies per ml	4.6 ± 1.6	4.70 ± 1.7	4.56 ± 1.6	0.713
Range (min.–max.),log10 copies / ml	2.60-7.5	2.60-7.4	2.60-7.5	--
Viral load group, copies per ml,%				
<500	35(36.5)	10(40)	25(35.2)	
501 – 10 000	22(22.9)	6(24)	16(22.5)	
10 001 – 250 000	10(10.4)	1(4)	9(12.7)	0.681**
>250 000	29(30.2)	8(32)	21(29.6)	
Missing	37			

**: Kruskal Wallis Test.

## Discussion

The burden of TB around the world remains alarming. Microscopy examination of sputum samples is recommended by the WHO as an essential step for patients suspected of pulmonary TB. Unfortunately, smear microscopy has been reported to have low sensitivity [33]. In particular, this technique has limited performance in immune-compromised individuals [34]. A number of studies report that pulmonary TB diagnosis can be achieved through the combination of clinical symptoms, CXR-examinations and smear microscopy [35–37].

As in many countries with limited resources where TB is endemic, the algorithm endorsed by the Congolese national TB control program emphasizes the use of these modalities. In our study, we found that this algorithm identifies less cases of PTB microscopy positive and PTB microscopy negative in HIV + individuals compared to HIV – individuals. It is important to keep in mind that mycobacterial culture or nucleic acid amplification tests are not available in our setting. This is a common limitation encountered in African countries. The true prevalence of pulmonary tuberculosis in our setting is thus not determined.

Our study showed that the rate of ZN smear positivity is higher in HIV - than in HIV + individuals. Given the well-known increased risk for pulmonary tuberculosis in HIV + individuals the data suggest that a number of HIV + individuals with pulmonary TB were not detected by ZN based microscopy [3, 6, 9]. Moreover the lower prevalence of CXR abnormalties in HIV + individuals also suggests that this diagnostic modality may be less sensitive in the HIV + population. Despite the limitation in methodology the present data thus suggest that the national diagnostic treatment algorithm may miss a substantial number of HIV + individuals with pulmonary tuberculosis. However, it is also important to note that patients who are smear-negative in our study, may not have TB. Against this background, we may speculate that many patients could be over-treated for TB.

In our study, the mean CD4/CD8 ratio was lower than 1, this indicates an increase of PTB among HIV patients, especially for negative smeargroup. Inability to properly diagnose pulmonary TB in the HIV infected population is hazardous to this immnuno-compromised population due to the well-known increased severity of pulmonary tuberculosis in TB-HIV co-infection. In addition there is a risk of immune-reconstitution inflammatory syndrome (IRIS) in patients on antiretroviral therapy with undiagnosed and those with untreated TB [4], this is unmasking TB IRIS. In other words, if TB remains undiagnosed in an HIV positive individual that starts antiretroviral therapy, there is a chance that the patient’s immune system may begin to recover but then respond to the TB infection with an overwhelming inflammatory response of patient [38].

Many studies have confirmed the superior effectiveness of culture methods when compared to smear microscopy [37, 39, 40]. To test TB diagnosis algorithms in resource-limited areas, the standard of care (evaluation through clinical symptoms, chest x-rays and smear microscopy) has been compared to confirmation of TB diagnosis through Mycobacteria Growth Indicator Tube (MGIT) technology which specifically detects and recovers mycobacteria. In a study conducted in Sub-Saharan Africa and South America, the standard of care method was less sensitive in detecting TB than the culture method [39]. In Kenya, [35] a cross sectional study was conducted to ascertain the performance of the 2007 World Health Organization (WHO) algorithm to diagnose PTB in HIV positive individuals. In this study, the use of culture increased significantly the proportion of confirmed TB cases. In 2010, WHO recommended the use of XpertMTB/RIF for HIV positive patients in order to detect *Mycobacterium tuberculosis* (Mtb) and rifampicin resistance [41]. GeneXpertMTB/RIF were reported to perform better than smear microscopy [42–44]. Recent studies have shown that the urinary LAM test improves the diagnosis of TB in HIV + individuals among those who are severely immunocompromised, however this test performance is high in the very sickest patients [45, 46].

The limit of the National algorithm for the diagnosis of TB infection is that no culture lab exists in the country. Moreover, with regard to high risk of HIV people to get TB infection, it would be very useful to test negative sputum samples with the WHO endorsed GeneXpert technology.

## Conclusion

In countries were TB and HIV are endemic, accurate and rapid diagnosis tools should be implemented. It is crucial to have access low-cost tools that are adapted to countries where resources are limited and to support health care providers with adequate training. This study showed that the current national treatment algorithm may adequately detect smear positive pulmonary TB in HIV- individuals.

In the present study, a consistent number of patients with smear-negative microscopy and negative radiology were found; therefore it is possible that many patients with negative smear pulmonary TB did not have TB. In order to avoid over-TB treatment, it appears urgent to use locally specific diagnostic methods such as culture or GeneXpert. However, our data also suggest that additional diagnostic modalities are required to diagnose TB in HIV + individuals, especially those with smear-negative pulmonary tuberculosis.
